# Do health checks improve risk factor detection in primary care? Matched cohort study using electronic health records

**DOI:** 10.1093/pubmed/fdv119

**Published:** 2016-10-17

**Authors:** Alice S. Forster, Caroline Burgess, Hiten Dodhia, Frances Fuller, Jane Miller, Lisa McDermott, Martin C. Gulliford

**Affiliations:** 1 Department of Primary Care and Public Health Sciences, King's College London, London, UK; 2 Public Health Directorate, London Boroughs of Lambeth and Southwark, London, UK; 3 Department of Public Health, London Borough of Lewisham, London, UK

**Keywords:** cardiovascular diseases, cardiovascular risk, deprivation, electronic health records, gender, health inequalities, primary care, screening

## Abstract

**Background:**

To evaluate the effect of NHS Health Checks on cardiovascular risk factor detection and inequalities.

**Methods:**

Matched cohort study in the Clinical Practice Research Datalink, including participants who received a health check in England between 1 April 2010 and 31 March 2013, together with matched control participants, with linked deprivation scores.

**Results:**

There were 91 618 eligible participants who received a health check, of whom 75 123 (82%) were matched with 182 245 controls. After the health check, 90% of men and 92% of women had complete data for blood pressure, total cholesterol, smoking and body mass index; a net 51% increase (*P* < 0.001) over controls. After the check, gender and deprivation inequalities in recording of all risk factors were lower than for controls. Net increase in risk factor detection was greater for hypercholesterolaemia (men +33%; women +32%) than for obesity (men +8%; women +4%) and hypertension in men only (+5%) (all *P* < 0.001). Detection of smoking was 5% lower in health check participants than controls (*P* < 0.001). Over 4 years, statins were prescribed to 11% of health -check participants and 7.6% controls (hazard ratio 1.58, 95% confidence interval 1.53–1.63, *P* < 0.001).

**Conclusion:**

NHS Health Checks are associated with increased detection of hypercholesterolaemia, and to a lesser extent obesity and hypertension, but smokers may be under-represented.

## Introduction

Cardiovascular diseases remain a major public health concern in the UK,^1^ as well as accounting for substantial healthcare costs.^2^ Since 2010, NHS Health Checks, a new programme for prevention of heart disease, stroke, kidney disease and diabetes has been introduced in England.^3^ The NHS Health Check programme aims to provide 5 yearly cardiovascular risk assessment for adults aged 40–74 years who have not yet developed cardiovascular disease and are not being treated for elevated cardiovascular risk. The programme has attracted controversy^4–6^ because older randomized trials suggest that health checks may increase healthcare utilization without yielding health benefits,^7^ while the results of more recent trials of cardiovascular risk reduction^8^ question whether universal cardiovascular risk assessment in primary care could prove cost-effective.

In the context of this controversy, evaluation of the NHS Health Check programme is important.^3^ Early evaluations suggested that there may have been initial problems including inconsistent organization and delivery of health checks^9^; low uptake of health checks following invitations to the programme^10^ and the lack of public^11^ and professional understanding of the purpose and design of the programme. In a study utilizing primary care electronic health records from the Clinical Practice Research Datalink (CPRD), we evaluated the yield of NHS Health Checks^12^ and found that for every 1000 men assessed, there were 205 smokers identified, 355 with hypertension and 633 with elevated cholesterol. For each 1000 women, there were 161 smokers, 247 with hypertension and 668 with elevated cholesterol identified. These results indicated a potential to improve the risk profile of participants in the NHS Health Check programme. Opportunistic screening and case finding are often undertaken in primary care but coverage may be uneven and there is potential for under-detection of high-risk states, potentially contributing to inequalities in data recording and risk factor detection. It is unclear to what extent the NHS Health Check programme adds to existing intervention through primary care. We aimed to compare risk factor detection and management among participants in the NHS Health Check programme, with matched controls who attended the same general practice but did not receive a heath check. As well as examining aggregate effects, we aimed to evaluate the impact of the health check programme at different levels of deprivation, and in men and women, in order to begin to understand the effect of the programme on health inequalities.

## Methods

### Data source and participant selection

Data for the study were obtained from the CPRD. The CPRD is a longitudinal database of anonymized electronic health records from general practices in the UK.^13^ The CPRD has recently been enriched by linkages to several data sources including indices of multiple deprivation (IMD) 2010 scores for selected general practices in England. The protocol was reviewed and approved by the CPRD Independent Scientific Advisory Committee (ISAC protocol 13_071A). Updated data were extracted from the October 2014 release of CPRD for 140 356 participants with a record of an NHS Health Check between 1 April 2010 and 31 March 2013, from 452 general practices in England, as reported previously.^12^ For the present analyses, we included all 106 784 participants registered with 334 general practices with IMD 2010 quintile for England, linked via the participant postcode, at the lower super-output area level. We excluded all patients who were ever treated with antihypertensive drugs (8531) or statins (1529) before the date of the check. We also excluded patients (4859) who did not have a full year of up to standard record before the date of the check and one patient whose check was after 31 March 2013. We also excluded patients with diabetes (227), coronary heart disease (2) or stroke (3) diagnosed before the check. There remained 91 618 participants who had a health check between 1 April 2010 and 31 March 2013, who had at least 12 months record, were never treated with antihypertensive drugs or statins, and were not diagnosed with diabetes, stroke or CHD before the check. For these 91 618 participants, eligible control participants were identified for 75 123 (82%) and these participants, and their matched controls, were included in analyses.

Control participants were selected who were eligible for NHS Health Checks but did not receive an NHS Health Check. Control participants might have been invited for a Health Check, but did not attend, or might not have been invited. Control participants were individually matched for general practice, gender and age (within ±2 years) with case participants who received an NHS Health Check. Control participants were assigned the same index date as the date of the check for matched case participants. Initially, there were 207 888 controls but after applying the same exclusion criteria as were applied to cases, there were 182 245 matched control participants analysed with linked IMD scores. Up to four controls were selected per case but this was only realized for 20% of cases, 28% of cases had three controls, 27% had two controls and 25% had only one control per case.

The effect of excluding health check participants, without eligible controls, had negligible effect on estimates: 89% of all eligible health check participants had all four risk factors measured [blood pressure, total cholesterol, smoking status and body mass index (BMI)], with mean systolic blood pressure 129.6 (SD 16.5) mmHg, compared with 91% with all four risk factor measures and systolic blood pressure 129.4 (16.5), in those matched with eligible controls.

### Main measures

Data were analysed for BMI, total cholesterol, systolic and diastolic blood pressure, smoking status^14^ and prescriptions for antihypertensive drugs and statins. Initially, we evaluated risk factor recording for NHS Health Check participants and controls, stratifying the results by gender and quintile of the IMD 2010 score. We evaluated the proportion of participants with values recorded for all four risk factors (blood pressure, total cholesterol, smoking status and BMI), as well as the proportion with values not recorded for each risk factor separately. The date of each risk factor record was evaluated with reference to the date of the Health Check (the date of the check was the reference date for the cases and their matched controls were also assigned this date of the check). We also evaluated risk factor detection, including the proportion of all patients with hypertension detected (blood pressure ≥140/90 mmHg), the proportion with hypercholesterolaemia detected (total cholesterol >5 mmol/l), the proportion with current smoking recorded and the proportion that were recorded as obese (BMI ≥30 kg/m^2^). We estimated the difference in proportions between health check participants and controls, together with *P*-values and 95% confidence intervals (CIs). However, because the sample was large, CIs were generally less than ±1% and are not shown. Adjusting for matched set using ‘svy’ commands in Stata version 13^15^ had negligible effect on precision of estimates. We also evaluated differences between men and women and between most and least-deprived deprivation quintiles. The difference between health check participants and controls in these differences, as measures of inequality, was evaluated. In the next stage of the analysis, we adopted a time-to-event framework with the date of check as the start date, to evaluate the onset of new prescriptions for antihypertensive therapy, stains and nicotine replacement therapy during up to 4 years from the date of the health check. The proportion of participants with these measures was estimated by year following the date of the check. Hazard ratios were estimated using the Cox proportional hazards model, allowing for clustering by matched set.

## Results

The analysis included 75 123 participants who received a health check between 1 April 2010 and 31 March 2013 and 182 245 matched controls who did not receive a health check. All participants had at least 12 months record before the index date and were never prescribed antihypertensive drugs or statins and never diagnosed with diabetes, coronary heart disease or stroke before the index date. The distribution of health check participants and controls by gender and deprivation quintile is shown in Table 1. The mean age was 54 years, 48% of participants were men, 15% of men and 14% of women were in the most deprived quintile for England. Controls were not matched for deprivation status, but nevertheless showed a similar distribution across deprivation quintiles as health check participants.

**Table 1:** Participants included in study by gender and deprivation quintile

*IMD2010 quintile*	*NHS health check participants*		*Controls*	
	**N* (%)*	*Age (mean, SD)*	**N* (%)*	*Age (mean, SD)*
Men				
Least deprived	8983 (25)	54 (9)	21 148 (23)	54 (9)
2	9109 (25)	54 (9)	21 604 (24)	54 (9)
3	6718 (18)	54 (9)	17 103 (19)	54 (9)
4	6226 (17)	53 (9)	16 410 (18)	53 (9)
Most deprived	5360 (15)	52 (8)	15 055 (16)	52 (9)
All	36 396	54 (9)	91 320	53 (9)
Women				
Least deprived	9378 (24)	54 (9)	21 853 (24)	54 (9)
2	9981 (26)	54 (9)	22 467 (25)	54 (9)
3	7248 (19)	54 (9)	17 285 (19)	54 (9)
4	6615 (17)	53 (9)	16 125 (18)	53 (9)
Most deprived	5469 (14)	52 (9)	13 195 (15)	52 (9)
All	38 727	54 (9)	90 925	54 (9)

Figures are frequencies (column percent) except where indicated.

Risk factor recording is contrasted for health check participants and controls in Table 2. Among participants who received a health check, 90% of men and 92% of women had complete records for four risk factors, including blood pressure, total cholesterol, smoking status and BMI. Among controls, 37% of men and 43% of women had four risk factor values recorded. The difference between health check participants and controls was 53% for men and 49% for women (both *P* < 0.001). In male control participants, there was a 9% difference between most and least deprived quintiles, in contrast to a 3% difference in health check participants yielding a net reduction in inequality of 6% (Table 2). No reduction in inequality was observed in women.

**Table 2:** Risk factor recording up to the NHS Health Check date by gender and deprivation quintile

*IMD quinitile*	*All four risk factors recorded^a^*		*Blood pressure not recorded*		*Total cholesterol not recorded*		*Smoking status not recorded*		*BMI not recorded*	
	*Men*	*Women*	*Men*	*Women*	*Men*	*Women*	*Men*	*Women*	*Men*	*Women*
NHS Checks										
Least deprived	8237 (91)	8744 (93)	20 (0)	3 (0)	646 (7)	583 (6)	4 (0)	3 (0)	132 (1)	67 (1)
2	8296 (91)	9204 (92)	18 (0)	6 (0)	700 (8)	707 (7)	4 (0)	2 (0)	139 (2)	84 (1)
3	5998 (89)	6651 (91)	14 (0)	6 (0)	642 (11)	587 (8)	6 (0)	0 (0)	99 (1)	61 (1)
4	5484 (88)	5891 (89)	13 (0)	8 (0)	655 (11)	671 (10)	3 (0)	3 (0)	107 (2)	65 (1)
Most deprived	4741 (88)	4989 (91)	17 (0)	3 (0)	533 (10)	435 (8)	3 (0)	2 (0)	109 (2)	51 (1)
All	32 756 (90)	35 479 (92)	81 (0)	26 (0)	3176 (9)	2983 (8)	20 (0)	10 (0)	586 (2)	328 (1)
Controls										
Least deprived	8668 (41)	9552 (44)	2087 (10)	632 (3)	11 530 (55)	11 722 (54)	1158 (5)	278 (1)	4199 (20)	2148 (10)
2	8306 (39)	9604 (43)	2333 (11)	696 (3)	12 309 (60)	12 238 (54)	1371 (6)	328 (1)	4608 (21)	2378 (10)
3	6200 (36)	7344 (43)	1933 (11)	570 (3)	10 193 (60)	9504 (55)	1069 (6)	253 (1)	3698 (22)	1738 (10)
4	5633 (34)	6843 (43)	2115 (13)	587 (4)	10 173 (62)	8891 (55)	1322 (8)	306 (2)	3650 (22)	1655 (10)
Most deprived	4818 (32)	5548 (42)	2267 (15)	608 (5)	9694 (64)	7353 (56)	1401 (9)	308 (2)	3677 (24)	1506 (11)
All	33 625 (37)	38 891 (43)	10 735 (12)	3093 (3)	53 899 (59)	49 708 (55)	6321 (7)	1473 (2)	19 832 (22)	9425 (10)
Difference_HC-Controls_	53%^b^	49%^b^	−12%^b^	−3%^b^	−50%^b^	−47%^b^	−7%^b^	−2%^b^	−20%^b^	−9%^b^
Difference^c^_inequality_	−6%	0%	−5%	−2%	−6%	0%	−4%	−1%	−3%	−2%

Figures are frequencies (percent of row totals shown in Table 1).

^a^Complete data for blood pressure, cholesterol, smoking and BMI.

^b^Test for difference of proportions, *P* < 0.001.

^c^Difference between health checks and controls for difference between most and least-deprived quintiles, negative values denoted reduced inequality.

Important differences in recording were noted for total cholesterol, with the proportion with no value recorded decreasing from 59 to 9% for men and from 55 to 8% in women. The net reduction in proportion with no value recorded was 50% for men and 47% for women, with a 6% reduction in deprivation inequality for men only. The proportion with BMI not recorded showed a net reduction of 20% in men and 9% in women, with 3% reduction in deprivation inequality in men and 2% in women. The net reduction in proportion with no blood pressure recorded was 12% in men and 3% in women, and for recording of smoking 7% in men and 2% in women. Reduction in deprivation inequality was greater for men being 5% for blood pressure recording and 4% for smoking records.

Risk factor records were more recent in health check participants (Table 3). Most values for blood pressure, smoking and BMI were recorded on the date of the check, with cholesterol values being recorded up to 3 weeks previously, consistent with a blood sample being taken for laboratory analysis prior to the check. In controls, the median time interval since the last systolic blood pressure record was 1.89 years, for total cholesterol 2.12 years, for smoking, 1.88 years and for BMI 3.12 years.

**Table 3:** Time since last risk factor measure for NHS Health Check participants and controls

*Measure*	*Time since last record*	
	*NHS Checks (days)*	*Controls (years)*
Blood pressure	0 (0–0)	1.89 (0.78–4.05)
Cholesterol	7 (1–22)	2.12 (0.88–4.20)
Smoking	0 (0–1)	1.88 (0.78–4.05)
BMI	0 (0–0)	3.12 (1.23–6.92)

Figures are median (interquartile range).

Table 4 shows the proportion of all participants with elevated risk factor values detected. A major effect of attending for the health check was an increase in the proportion of participants with hypercholesterolaemia detected from 26% in men and 30% in women, among controls, to 59% in men and 62% in women who received a health check. This represented a net increase of 33% for men and 32% for women. Deprivation inequality reduced by 3% in men but increased by 3% in women. There were smaller increases in the proportion with obesity detected from 15% in men and 19% in women controls, to 23% in men and women after the health check. This represented a net increase of 8% in men and 4% in women, with deprivation inequality in recorded obesity increasing in both men and women. The proportion with high blood pressure detected increased from 31 to 36% in men and remained at 24% in women, a net increase of 5% in men and 0% in women. Deprivation inequality in detected hypertension increased by 6% in men. The proportion with smoking detected was 5% higher in control participants, possibly suggesting that a higher proportion of non-smokers attended for health checks.

**Table 4:** Risk factor detection at the NHS Health Check by gender and deprivation quintile

*IMD quinitile*	*Hypertension detected ≥140/90 mmHg*		*Hypercholesterolaemia detected >5 mmol/l*		*Current smoking detected*		*BMI ≥30 kg/m^2^ detected*	
	*Men*	*Women*	*Men*	*Women*	*Men*	*Women*	*Men*	*Women*
NHS Checks								
Least deprived	3231 (26)	2153 (23)	5559 (62)	5973 (64)	1216 (14)	915 (10)	1806 (20)	1710 (18)
2	3278 (36)	2420 (24)	5510 (60)	6296 (63)	1557 (17)	1244 (12)	2029 (22)	2021 (20)
3	2605 (39)	1938 (27)	3959 (59)	4538 (62)	1410 (21)	1133 (16)	1640 (24)	1673 (23)
4	2221 (36)	1545 (23)	3499 (56)	3892 (59)	1647 (26)	1402 (21)	1580 (25)	1791 (27)
Most deprived	1848 (34)	1289 (24)	2966 (56)	3169 (58)	1945 (36)	1606 (29)	1414 (26)	1741 (32)
All	13 183 (36)	9345 (24)	21 523 (59)	23 868 (62)	7775 (21)	6300 (16)	8469 (23)	8936 (23)
Controls								
Least deprived	6447 (30)	5109 (23)	6281 (30)	6646 (30)	3530 (17)	2756 (13)	2953 (14)	3286 (15)
2	6924 (32)	5609 (25)	6006 (28)	6854 (31)	4598 (21)	3690 (16)	3116 (14)	3778 (17)
3	5505 (32)	4375 (25)	4416 (26)	5222 (30)	4335 (25)	3585 (21)	2796 (16)	3418 (20)
4	4790 (29)	3863 (24)	3882 (24)	4577 (28)	5336 (32)	4341 (27)	2582 (16)	3634 (23)
Most deprived	4272 (28)	3107 (24)	3181 (21)	3612 (27)	6042 (40)	4699 (36)	2477 (16)	3272 (26)
All	27 938 (31)	22 063 (24)	23 766 (26)	26 911 (30)	26 841 (26)	19 071 (21)	13 924 (15)	17 489 (19)
Difference_HC-Controls_	+5%^a^	0%^b^	+33%^a^	+32%^a^	−5%^a^	−5%^a^	+8%^a^	+4%^a^
Difference_inequality_^c^	+6%	0%	−3%	+3%	−1%	−4%	+4%	+3%

Figures are frequencies (percent of total with values recorded).

^a^Test for difference of proportions, *P* < 0.001 or ^b^*P* = 0.420.

^c^Difference between health checks and controls for difference between most and least-deprived quintiles, negative values denoted reduced inequality.

The median duration of follow-up was 2.2 (IQR 1.4–2.8) years for health check participants and 2.2 (1.6–3.0) years for controls. Table 5 shows changes over time in statin and antihypertensive drug prescribing following the health check. New antihypertensive prescribing was initiated for 12.4% of health check patients and 11.2% of controls in the first 4 years following the check. This small difference was statistically significant but of very small magnitude. New statin prescriptions were initiated for 11.0% of health check patients and 7.6% of controls, hazard ratio 1.58 (95% CI 1.53–1.63).

**Table 5:** Prescription of antihypertensive drugs and statins in 4 years following the health check

*Year following check*	*New antihypertensive drug prescribing*		*New statin prescribing*	
	*NHS Checks*	*Controls*	*NHS Checks*	*Controls*
First	4.4 (4.3–4.6)	4.2 (4.1–4.2)	4.9 (4.7–5.0)	2.6 (2.5–2.6)
Second	7.0 (6.9–7.2)	6.8 (6.6–6.9)	6.9 (6.7–7.1)	4.3 (4.2–4.4)
Third	9.8 (9.7–10.1)	9.1 (8.9–9.3)	8.8 (8.6–9.1)	6.1 (5.9–6.2)
Fourth	12.4 (12.0–12.9)	11.2 (11.0–11.5)	11.0 (10.6–11.4)	7.6 (7.4–7.9)
Hazard ratio^a^ (95% CI)	1.06 (1.03–1.10)		1.58 (1.53–1.63)	
*P*-value	<0.001		<0.001	

Figures represent the cumulative proportion (95% CI) of participants prescribed.

^a^Adjusted for age, gender and deprivation quintile.

## Discussion

### Main findings of this study

A major effect of the NHS Health Check programme is to increase recording of risk factor values in primary care. More than 90% of participants in the programme had complete values for blood pressure, total cholesterol, smoking and BMI recorded at the date of the check. In matched control participants, who did not have a health check, some 40% or fewer participants had records of all four risk factor values and those values available were less recently recorded. Increases in risk factor recording were associated with quantitatively important reductions in inequalities in recording associated with gender and deprivation quintile. Reductions in inequality were generally greater in men than in women, possibly because women consult more regularly in primary care. Increased recording was also associated with improved risk factor detection with increases in the proportion of participants recognized to have hypercholesterolaemia, obesity and to a lesser extent hypertension. A small proportion of cases did not have cholesterol measurements recorded by the date of the check. We noted that some patients had cholesterol values recorded after the date of the check but we did not include these as there was no clear cut-off date that would allow attribution to the check. A higher proportion of controls were recorded as smokers, which suggests that there is lower participation in the NHS Health Check programme by smokers. In contrast to the findings for risk factor recording, the general tendency was for deprivation inequalities in risk factor detection to be increased following the health check, with greater inequality observed for obesity in men and women, for hypertension in men and for hypercholesterolaemia in women, consistent with known social gradients in these risk factors.^16^ Health checks may have a role to play in identifying individuals at higher risk from lower socioeconomic groups, but this requires programme uptake to be maintained in these groups. The clinical response was relatively modest with slightly higher prescribing of statins among patients who attended for health checks, but only very small differences observed for antihypertensive prescribing.

### What is already known on this topic?

A number of previous studies of the NHS Health Checks have explored the problem of low uptake of invitations to participate in the programme,^10,17^ exploring influences on the decision to attend for a check^11^ and suggesting and evaluating methods to increase uptake of health checks.^18,19^ Other studies have explored individual patients' responses to participation in the programme^20^ and the potential of the programme to influence cardiovascular risk.^21,22^ A study in Warwickshire evaluated detection of cardiovascular disease through the NHS Health Check programme.^23,24^ Caley *et al*.^23^ reported on 38 general practices that provided health checks and 41 control practices that did not. They found that >16 000 checks were associated with detection of >1000 previously undiagnosed cases of disease. However, there were no differences in the prevalence of diabetes, hypertension, coronary heart disease, chronic kidney disease and atrial fibrillation in practices providing NHS Health Checks compared with control practices. Our results suggest that there will usually be a substantial net increase in the proportion of participants with elevated risk factor status detected. However, this increase is mainly observed for elevated cholesterol, with more modest increases observed for hypertension and obesity, with the main benefit being in men. It appears likely that under-representation of smokers may prevent any additional net yield of additional smokers detected.

### What this study adds

This study shows that there are improvements in risk factor recording in primary care for participants who have received an NHS Health Check, when compared with participants who only receive opportunistic screening. We show that this improvement is associated with reduced gender differences in risk factor recording and generally smaller socioeconomic differentials in risk factor recording. This leads to increased detection of elevated risk factor status, with the exception of smoking, revealing greater deprivation inequalities in these measures than are apparent in the absence of a health check. Whether the health check programme has an impact on inequalities may depend on the balance between inequalities in uptake and inequalities in outcomes. We caution that inequalities in health check uptake were not addressed directly through this study and these may be important. The major impact of the programme is to lead to substantially increased detection of hypercholesterolaemia, with smaller increases in detection of obesity and hypertension. During a maximum of 4-year follow-up, statin prescribing increased to 11.0% of health check participants compared with 7.6% of controls. This may be interpreted as providing reassurance that the health check programme may not lead to widespread prescribing of lipid-lowering drugs. The use of statins for primary prevention remains controversial^25^ and these drugs are not always well tolerated. Ultimately, the effectiveness of the programme will depend on the impact of the interventions prescribed following the health check. Further monitoring of the longer term effectiveness of the programme needs to consider the impact of prescribed behavioural and pharmacological interventions. Longer term follow-up is also required in order to evaluate changes in risk and cardiovascular and mortality outcomes.

### Strengths and limitations of this study

The study employed a large sample with 75 123 participants who received a health check and 182 245 matched controls drawn from 334 general practices in England. The CPRD population is generally considered to be representative of the resident population in the UK.^13^ Data from CPRD presently derive from general practices that use the Vision Practice system but data from other practice systems are now being incorporated into CPRD and might be available for future analyses. In the present study, we used a subset of CPRD general practices that provided linked IMD score data. We included 82% of eligible participants in the analysis. The main reason for loss of participants was a lack of suitable controls. This arose because, owing to the size and complexity of the CPRD database, it was necessary to apply exclusion criteria to controls after their selection from CPRD. Data from CPRD have been the subject of many validation studies,^26^ in particular smoking prevalence estimates from CPRD have been found to be similar to those obtained from the Health Survey for England.^14^ There were minor differences in measures between this report and our previous study^12^ that were likely to have arisen through using a dataset that was updated to October 2014. The control sample might have included participants who were invited for a health check but did not attend, whereas cases were invited and did attend. The reasons why controls did not attend for a Health Check might have been associated with recording of their measurements in general practice.

### Conclusions

Receipt of NHS Health Checks is associated with increased detection of hypercholesterolaemia, and to a lesser extent obesity and hypertension, but smokers may be under-represented. However, the effectiveness and longer term outcomes of behaviour change approaches to risk reduction following a health check remain uncertain and require further research, leading to the development of more effective intervention strategies. Longer term follow-up is also required in order to evaluate changes in risk and cardiovascular and mortality outcomes.

## Funding

This work was supported by the Guy's and St. Thomas’ Charity (grant no. G100702).
